# Inhibitor SBFI26 suppresses the malignant progression of castration-resistant PC3-M cells by competitively binding to oncogenic FABP5

**DOI:** 10.18632/oncotarget.16055

**Published:** 2017-03-09

**Authors:** Waseem Al-Jameel, Xiaojun Gou, Shiva S. Forootan, Majed Saad Al Fayi, Philip S. Rudland, Farzad S. Forootan, Jiacheng Zhang, Philip A. Cornford, Syed A. Hussain, Youqiang Ke

**Affiliations:** ^1^ Molecular Pathology Laboratory, Department of Molecular and Clinical Cancer Medicine, Liverpool University, Liverpool, L3 9TA, United Kingdom; ^2^ Sichuan Antibiotics Industrial Institute, Chengdu University, Chengdu 610081, China; ^3^ Department of Biochemistry, Liverpool University, Liverpool, L69 3GA, United Kingdom

**Keywords:** SBFI26, FABP5, CRPC, PPARγ, metastasis

## Abstract

Castration resistant-prostate cancer is largely impervious to feather hormonal therapy and hence the outlook for patients is grim. Here we use an approach to attach the recently discovered Achilles heel. The experimental treatment established in this study is based on the recent discovery that it is the FABP5-PPARγ-VEGF signalling axis, rather than the androgen receptor pathway, played a dominant role in promoting the malignant progression of castration resistant prostate cancer cells. Treatments have been established in mice by suppressing the biological activity of FABP5 using a chemical inhibitor SBFI26. The inhibitor significantly suppressed the proliferation, migration, invasiveness and colony formation of PC3-M cells *in vitro*. It also produced a highly significant suppression of both the metastases and the primary tumours developed from cancer cells implanted orthotopically into the prostate glands of the mice. The inhibitor SBFI26 interferes with the FABP5-PPARγ- signalling pathway at the initial stage of the signal transduction by binding competitively to FABP5 to inhibit cellular fatty acid uptake. This avoids the fatty-acid stimulation of PPARγ and prevents it activating the down-stream regulated cancer-promoting genes. This entirely novel experimental approach to treating castration- resistant prostate cancer is completely different from current treatments that are based on androgen-blockade therapy.

## INTRODUCTION

Prostate cancer is an important cause of mortality in men, mainly in countries where a high dietary ratio of fatty acids is consumed [1]. Androgen deprivation therapy (ADT) is the first line treatment for advanced prostate cancer and it is initially effective. However, in nearly all cases the disease eventually relapses within 2–3 years, with a lethal castration-resistant prostate cancer (CRPC); this cannot be effectively treated with ADT anymore [2]. Thus, identification of new targets for novel effective therapeutic approaches is urgently needed for the effective treatment of CRPC patients. The CRPC cells overexpress fatty acid synthase (FASN) and acetyl-CoA carboxylase (ACC) which are key enzymes involved in synthesis of fatty acids [3–5]. Fatty acids are not only active components of many biological processes, but also are essential signal molecules in pathways involved in prostate cancer progression, and hence can increase the risk of advanced prostate cancer [6, 7] and play an important role in carcinogenesis and metastasis of cancer cells [8].

Fatty acid-binding protein 5, or FABP5, is a 15kDa cytosolic protein binding with a high affinity to medium and long chain fatty acids [9]. After its crucial activity in promoting malignant progression in cancer cells was initially demonstrated [10, 11], increased FABP5 expression in archival prostate cancer tissues is found to be significantly associated with a reduced patient survival time. Thus it is a valuable prognostic factor [12]. Moreover, investigations in the past few years established that there is a novel fatty acid-initiated signalling pathway leading to malignant progression of prostatic cancer cells. Thus when FABP5 expression is increased, excessive amounts of fatty acids are transported into the nucleus, where they act as signalling molecules to stimulate their nuclear receptor PPARγ. The activated PPARγ then modulates expression of its down-stream regulatory genes which finally lead to enhanced tumour expansion and aggressiveness caused by an overgrowth of cells with increased angiogenesis and reduced apoptosis [13]. Recently, it was suggested that the FABP5-PPARγ-VEGF signalling transduction axis, rather than androgen receptor (AR)-modulated signal transduction pathway, that is the dominant signalling route in promoting malignant progression of CRPC cells [7]. Although the molecular mechanism involved in cancer-promoting activity of FABP5 has been extensively studied, it was not clear whether the CRPC can be treated by suppressing the biological activity of the oncogenic FABP5. The availability of a highly effective inhibitor is an important first step. Inhibition of FABP5 activity was shown to be effective for treatment of inflammatory and metabolic diseases by chemically synthesized inhibitors, e.g. BMS309403 [14–17]. Recently developed FABP5 inhibitors, approximately 50% inhibitory effect of BMS309403, were originally used effectively as analgesic and anti-inflammatory agents in mice [18–20]. These included SBFI26 (α-truxillic acid 1-naphthyl mono-ester). SBFI26 was, in fact, the active component of a Chinese herbal medicine (Incarvillea sinensis) which was used to treat pain and rheumatism in humans in Chinese traditional medicine since hundreds of years ago [21, 22].

In a strategy to develop anti-inflammatory and anti-nociceptive reagents by targeting fatty acid protein anandamide transporters, SBFI26 was used to increase the brain anandamide levels and thus to produce analgesia effect [18, 19]. Although SBFI26 has been used as an inhibitor of FABP5 in anandamide transportation in brain, its possible effect on cancerous diseases was not known. In this study, we targeted the FABP5-related signalling pathway to treat CRPC in mice by using the SBFI26 to suppress the biological activity of FABP5 and to cut off the FABP5-related signalling transduction chain in CRPC cells. This is an entirely novel experimental approach to treat CRPC and is completely different from current treatments that are based on androgen-blockade therapy.

## RESULTS

### Identification of lead inhibitor of FABP5 from a group of chemical compounds

**Figure 1:**
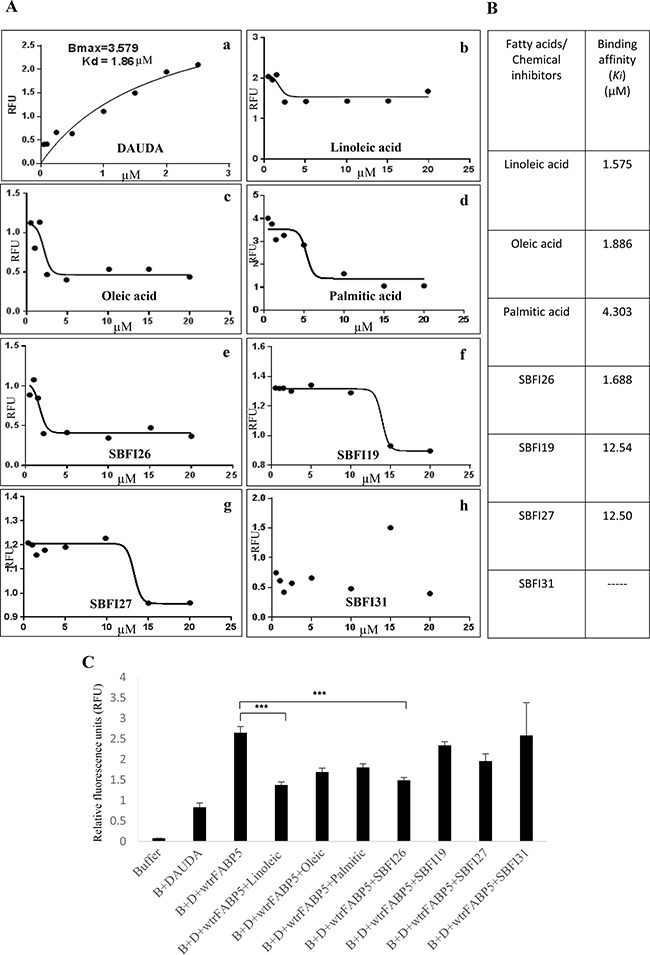
Detection of binding affinities of 4 candidate compounds to wtrFABP5 with DAUDA displacement assay to identify the lead chemical inhibitor of FABP5 (**A**) Chart records of binding affinity analysis of 3 different fatty acids and 4 different candidate chemical inhibitors of FABP5. **a**) Titration curve of DAUDA binding to wtrFABP5. Fixed amounts (3 μM) of wtrFABP5 were incubated with increasing concentrations of DAUDA (0.4–3 μM). For calculation of the dissociation constant *K*_d_ values, the excitation and emission used was 345 and 530 nm, respectively and the fluorescence data was normalized to the peak fluorescent intensity for each experiment and data of samples without protein was subtracted. The data was fitted by nonlinear regression techniques using GraphPad Prism software to a saturation binding curve model to estimate the apparent dissociation constant (*K*_d_) and maximal fluorescence intensity (*B*_max_). The apparent dissociation constant (*K*_d_) for DAUDA to wtrFABP5 was calculated to be 1.86 ± 0.16 μM. (b, c, d) Inhibition constant *K*_i_ (binding affinity) of Linoleic, Oleic and Palmitic acids binding to wtrFABP5. The *K*_i_ was measured to determine the potency of binding of these fatty acids with wtrFABP5 by evaluating their ability to displace DAUDA. The data were collected by displacement of 2 μM DAUDA from 3 μM wtrFABP5 in the presence of different concentrations of each fatty acid (0.5–20 μM). All data were fitted to a one site binding affinity model by non-linear regression techniques using GraphPad Prism software to estimate the binding affinity. The *K*_i_ of each ligand was determined using the equation *Ki = IC*_50_/1+ (DAUDA concentration /*K*_d_). The binding affinity of Linoleic acid (*K*_i_ = 1.57 μM) was higher than that of Oleic and Palmitic acids (*K*_i_ = 1.88 and 4.30 μM, respectively). (e, f, g) Inhibition constant *K*_i_ of SBFI26, SBFI19, SBFI27, and SBFI31 to wtrFABP5. The data were collected by displacement of 2 μM DAUDA from 3μM wtrFABP5 in the presence of different concentrations (0.5–20 μM) of each chemical compound. All data were fitted to a one site binding affinity model by non-linear regression techniques using GraphPad Prism software to estimate the binding affinity. The binding affinity of SBFI26 (*K*_i_ =1.68 μM) was the highest amongst the 4 compounds. (**B**) *K*_i_ values of 3 different fatty acids and 4 different candidate chemical inhibitors. (**C**) Fluorescence intensity of displacement of 2 μM DAUDA binding from 3μM wtrFABP5 in the presence of 10 μM of 3 different fatty acids and 4 different candidate chemical inhibitors. The value of fluorescence intensity produced by the buffer and DAUDA plus wtrFABP5 was set as control. The results (mean ± SE) were obtained from 3 separate experiments (2-tailed unpaired Student’s *t* test, ***P < 0.0001).

The inhibition constants (*K*_i_) of 3 natural fatty acids (linoleic, oleic, palmitic acid) and 4 chemical compounds (SBFI26, SBFI19, SBFT27, SBFI31) which inhibited 50% of the binding of fluorescent substrate DAUDA to wtrFABP5 were measured to identify the most potent inhibitor (Figure 1A). The *K*_d_ of DAUDA- wtrFABP5 was 1.86 ± 0.16 μM (Figure 1A/a). The calculated *K*_i_ (μM) values (Table B) of linoleic, oleic and palmitic acid were 1.58 ± 0.14, 1.89 ± 0.18 and 4.30 ± 0.4, respectively (Figure 1A/b, c, d). Thus linoleic acid had the strongest binding affinity for wtrFABP5. The most potent compound to bind wtrFABP5 was SBFI26 (*K*_i_ = 1.69 ± 0.15 μM), whose affinity was about 7.4- times higher than those of SBFI19 and SBFI27 (*K*_i_ = 12.54 ± 2.25 and 12.50 ± 2.07 μM, respectively). The *K*_i_ of SBFI31 did not converge (Figure 1A/e, f, g, h). The binding affinity (*Ki*) of the lead compound (SBFI26) for wtrFABP5 was similar to that of the best- binding fatty acid (linoleic acid). DAUDA assay was performed to ascertain the degree of displacement of DAUDA from wtrFABP5 by the chemical inhibitors and the results are shown in Figure 1C. The relative level of fluorescence intensity of wtrFABP5 with DAUDA was 2.65 ± 0.14. This was reduced to 1.37 ± 0.07, 1.68 ± 0.09 and 1.80 ± 0.08 after adding linoleic, oleic and palmitic acids, respectively, to the complex. After SBFI26, SBFI19, SBFT27, and SBFI31 were added to the complex, the intensity was reduced to 1.48 ± 0.06, 2.33 ± 0.08, 1.95 ± 0.17 and 2.58 ± 0.79, respectively. Linoleic acid and SBFI26 produced highly significant reductions in fluorescence intensities (Student’s *t* test, *P* < 0.0001).

### Inhibitory effect of SBFI26 on malignant characteristics of PC3-M cells

**Figure 2:**
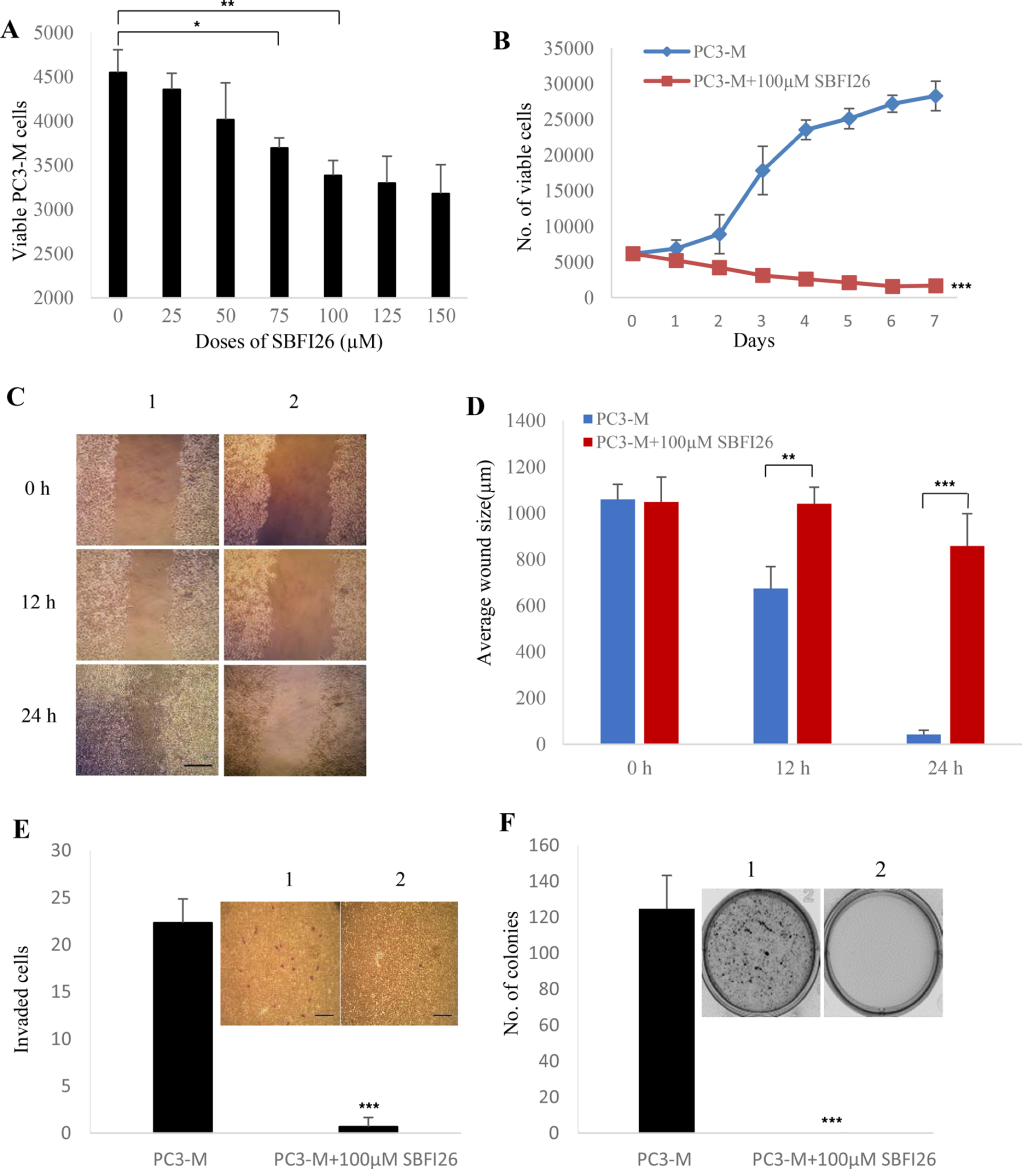
Inhibitory effect of SBFI26 on proliferation, migration, invasion and anchorage-independent growth of the androgen-independent PC3-M prostate cancer cells (**A**) Determination of the optimal inhibitory concentration of SBFI26 at which the maximum suppression of cell growth is achieved. MTT assay was performed to measure the viable PC3-M cell numbers of the control (untreated) and those treated with different concentrations of SBFI26 for 24 h. (**B**) Inhibitory effect of 100 μM SBFI26 on proliferation of PC3-M cells over the 7day experimental period. (**C**) Representative photos of the wound healing assay. PC3-M cells were grown in 6-well plates to form a monolayer. Scratches were made using 1 mL sterile pipette tip. Cell migration capacity was measured by the reduction in wound size in control (1) and in cultures treated with 100 μM SBFI26 (2) observed at 0, 12 and 24 hours after treatment. The scale bar is 250 μm. (**D**) Average wound sizes (μm) of the control PC3-M and cultures treated with 100 μM SBFI26 observed at 0, 12 and 24 hours after treatment. Data was collected by measuring image of the wound space and analyzed by ImageJ software (National Institutes of Health). (**E**) Number of invading cells from the control PC3-M cells (1) and cultures treated with 100 μM SBFI26 (2) for 24 h after different treatments. Results (mean ± SE) are obtained from three separate measurements. Scale bar is 250 μm. (**F**) Colonies produced by the control PC3-M cells (1) and cultures treated with 100 μM SBFI26 (2) in soft agar 2 weeks after the different treatments. Results (mean ± SE) are obtained from three separate plates in each treatment. The inserted picture was a representative plate from each of the 3 treatments. All *in vitro* results were subjected to 2-tailed unpaired Student’s *t* test and *P < 0.05; **P < 0.001; ***P < 0.0001.

Results of the inhibitory effect of SBFI26 on malignant characteristics of the PC3-M prostate cancer cells are shown in Figure 2. Cytotoxicity tests showed that treatment with SBFI26 significantly suppressed viability of PC3-M cells in a concentration- dependent pattern. Maximum suppression was produced at 100 μM for SBFI26; further increase in doses did not produce any further significant suppression. When treated with this optimal dose, cell numbers were significantly reduced by 26% (Student’s *t* test, *P* < 0.001) (Figure 2A). When tested using a MTT assay, 100 μM SBFI26 significantly reduced the proliferation rate of PC3-M cells by 17-times (Student’s *t* test, *P* < 0.0001) (Figure 2B). When tested in a cell migration assay (Figure 2C), treatments with 100 μM SBFI26 produced only 19% reduction in wound size in 24h. This treatment significantly suppressed the migration rates of PC3-M cells (Student’s *t* test, *p* < 0.0001), leading only to small changes in wound gaps for the treated group compared to an almost complete gap closure (94%) for the control (Figure 2D). When tested in an invasion assay, the mean numbers of invaded cells from the control and the PC3-M cells treated with SBFI26 were 22 ± 3 and 1 ± 1, respectively, representing a highly significant suppression of invasion by 95.5% (Student’s *t* test, *P* < 0.0001) (Figure 2E). Further tests in soft agar showed that the number of colonies formed after 2 weeks by control PC3-M cells and PC3-M cells treated with SBFI26 were 124 ± 18 and 0, respectively, representing a highly significant inhibition by 100% (Student’s *t* test, *p* < 0.0001) (Figure 2F).

### Effect of SBFI26 on tumourigenicity and metastatic ability of PC3-M cells in mouse prostate gland

**Figure 3:**
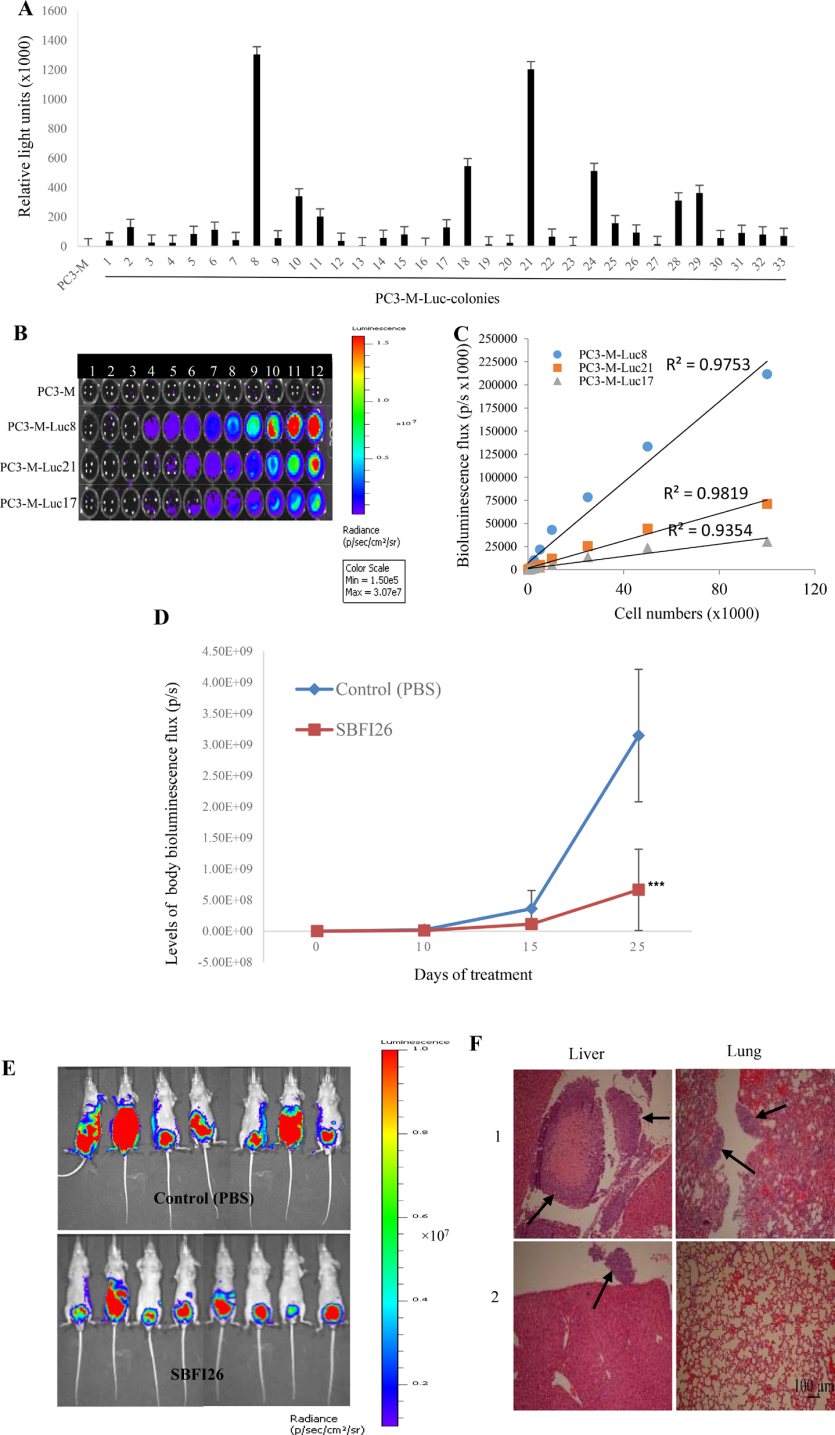
Inhibitory effect of SBFI26 on tumorigenicity and metastatic ability of PC3-M cells implanted orthotopically into the prostate gland of the nude mouse (**A**) Establishment of stable PC3-M colonies expressing strong bioluminescence signals by pGL4.50 [*luc2*/CMV/Hygro] vector transfection. Relative light units (mean ± SE) of the PC3-M parental cells and 33 colonies derived from PC3-M cells were obtained from 3 separate measurements. Individual colonies were isolated by ring cloning and 3 colonies that stably-expressed the highest bioluminescence signals were identified using D-luciferin (Promega) with a Varioskan Flash Reader (Thermo Scientific). (**B**) Detailed observation of the intensities of the bioluminescence images of the serially-diluted (20-100000) parental PC3-M cells and 3 representative PC3M-Luc transfectants. Association of the luminescence intensity with the number of cells was assessed by an IVIS imaging system (Perkin Elmer). The color bar on the right indicates the signal intensity range (photons/second/cm^2^). (**C**) Correlation between the bioluminescence flux intensity (photons/second) and the number of cells derived from 3 different PC3-M-Luc colonies. (**D**) Whole body tumor bioluminescence flux produced by each group of nude mice after orthotopic implantation of luciferase-labelled PC3-M cells and treated with PBS (control), SBFI26 (1 mg/kg) for 25 days. Values were plotted as mean ± SE (error bars) (*n* = 8); the difference between the control and each of the testing groups was assessed by two-tailed unpaired Student’s *t* test ***P < 0.0001. (**E**) Ventral bioluminescence images of primary tumors and metastases in all groups of experimental mice 25 days after treatment. The color bar on the right indicates the signal intensity range (photons/second/cm^2^). (**F**) Representative photomicrographs of detection of liver and lung metastases (arrows) from mice which received injection of PBS (1) and SBFI26 (2). Sections of tissues were stained with H&E. Magnification, ×10 and scale bar is 100 μm. All animal work was performed in accordance with UKCCCR guidelines under Home Office License PPL40/2963.

PC3-M cells were stably transfected with the luciferase vector and the 2 transfectant colonies that generated high bioluminescence signals were picked and named PC3-M-Luc8 and 21, respectively (Figure 3A). Further measurement with the IVIS image system showed that PC3-M-Luc8 produced the highest level of bioluminescence signal (Figure 3B) and there was a correlation between total flux and the number of labelled cells (R^2^ = 0.98) (Figure 3C). Luciferase-labelled PC3-M-Luc8 were implanted orthotopically into the dorsolateral side of the prostate of each of 2 groups of nude mice that were then intraperitoneally injected daily with PBS and SBFI26, respectively, for 25 days. At day 25, there was a massive decrease in bioluminescence signal (p/sec/cm^2^) in SBFI26 (6.66 × 10^8^) treated group in comparison with the control (31.5 × 10^8^). On the basis of bioluminescence, our results showed about 4.9-fold suppression in tumour masses by SBFI26 over those of control group (Student’s *t* test, *P* < 0.0001) (Figure 3D). In the control group, 7/7 (100%) mice produced metastases. In the group treated with SBFI26, 4/8 (50%) of mice produced visceral metastasis. A suppression of 50% in metastasis incidence was seen when compared to the control group (Figure 3E). Histological staining showed that all mice developed metastases in the control group, mainly in the liver and lung. In the SBFI26 treated group, half of the mice developed liver metastases with no metastasis in the lung. One representative stained slide from each group/organ is shown in Figure 3F.

### SBFI26 inhibited tumourigenicity of PC3-M cells in nude mice in a similar way to PPARγ antagonist

**Figure 4:**
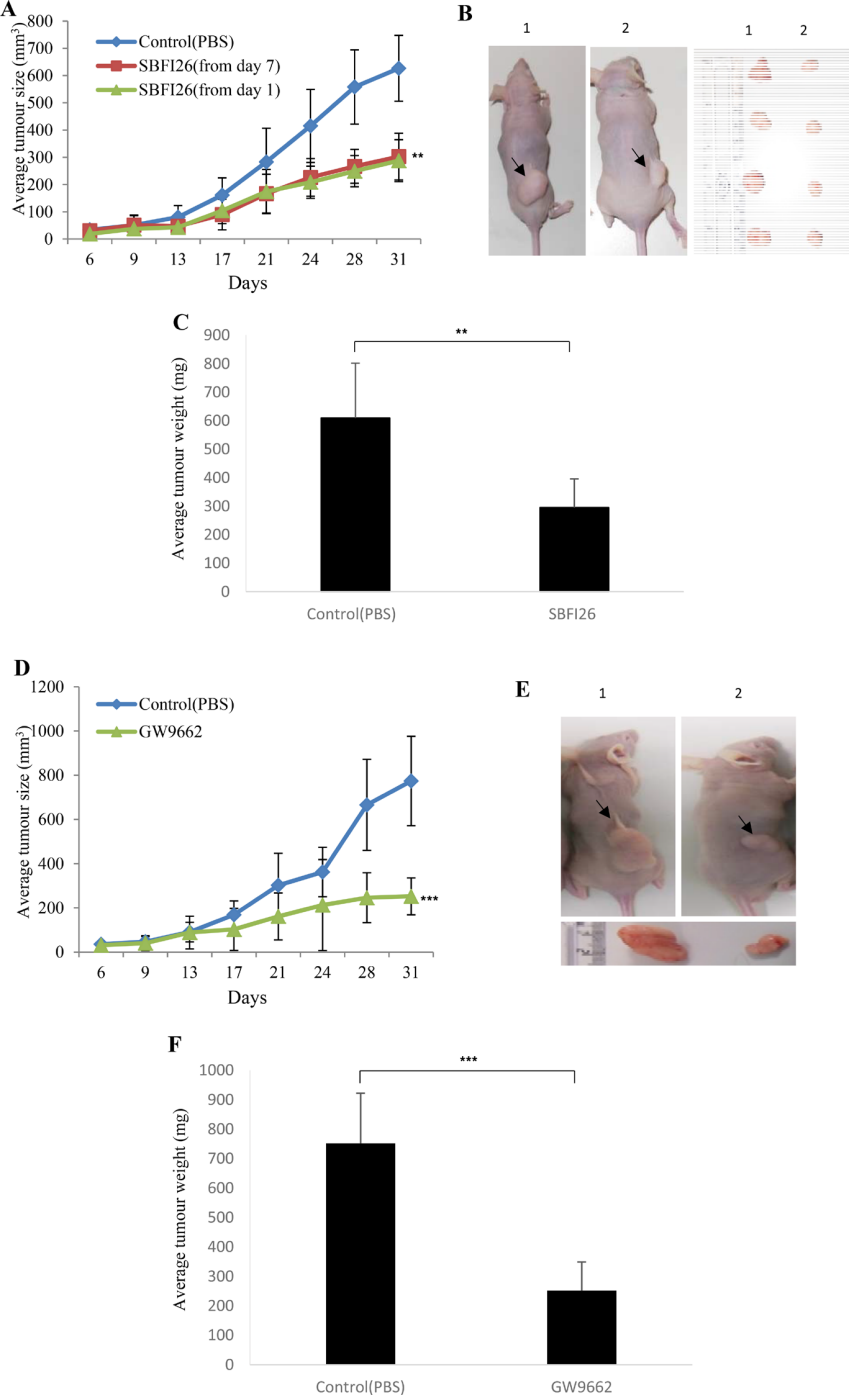
Effect of SBFI26 or GW9662 on tumorigenicity in prostate cancer xenograft mice (**A**) Average volume of tumors produced by each group of male nude mice after subcutaneous inoculation of PC3-M cells (2 × 10^6^) and treated with PBS (control) or SBFI26 (1 mg/kg) for 31 days; started on day 1 and day 7 after inoculation. Values are plotted as mean ± SE (error bars) (*n* = 8); difference between the control group and the experimental groups were assessed by 2-tailed unpaired Student’s *t* test, **P < 0.001. (**B**) Representative mouse and its corresponding tumors from control (1) and SBFI26 treated (2) groups. (**C**) Average weight (mg) of tumours from control and SBFI26 treated groups of mice. Values were plotted as mean ± SE (error bars). The differences between the control and the experimental groups were assessed by 2-tailed unpaired Student’s *t* test **P < 0.001. (**D**) Average volume of tumors produced by each group of male nude mice after subcutaneous inoculation with PC3-M cancer cells and treated with PBS (control) and PPARγ antagonist (GW9662;1 mg/kg) for 31 days. Values were plotted as mean ± SE (error bars) (*n* = 5); differences between the control and the experimental groups were assessed by 2-tailed unpaired Student’s *t* test ***P < 0.0001. (**E**) Representative mouse and its corresponding tumor from each of the control (1) and GW9662 (2) groups. (**F**) Average weight (mg) of tumours in the control and experimental groups of mice. Values were plotted as mean ± SE (error bars). Differences between the control and the experimental groups were assessed by 2-tailed unpaired Student’s *t* test ***P < 0.0001.

PC3-M cells were inoculated into the right flank of nude mice and the FABP5 inhibitor SBFI26 was injected subcutaneously into the flank of the mice to compare its anticancer effect with that of PPARγ antagonist (Figure 4). Although remarkable suppression of tumour growth was found in mice treated with the inhibitor (Figure 4B), no significant difference in treatment effect was found when inhibitor was applied from day 1 or from day 7 after the inoculation. On termination, average volumes of tumours in the group treated with SBFI26 was 302 ± 86 mm^3^, compared to 627 ± 120 mm^3^ in the control group; significant suppressions of 52% (Student’s *t* test, *p* < 0.001) (Figure 4A and 4B). When tumours were weighed on termination, the difference between control and treated group was similar to that measured by tumour volume (Figure 4C). To study possible suppression by PPARγ antagonist GW9662, mice were injected with PBS and with GW9662, respectively, from day 7 after inoculations (Figure 4E). Compared to the average size of tumours (774 ± 202 mm^3^) in control group, the average size of tumours in the group treated with GW9662 was reduced to only 252 ± 84 mm^3^, a highly significant suppression by 67% (Student’s *t* test, *p* < 0.0001) (Figure 4D). When tumour weight was measured at autopsy, the difference between control and the treated group was similar to that measured by tumour volume (Figure 4F).

### SBFI26 inhibited fatty acid uptake of FABP5 in PC3-M cells

**Figure 5:**
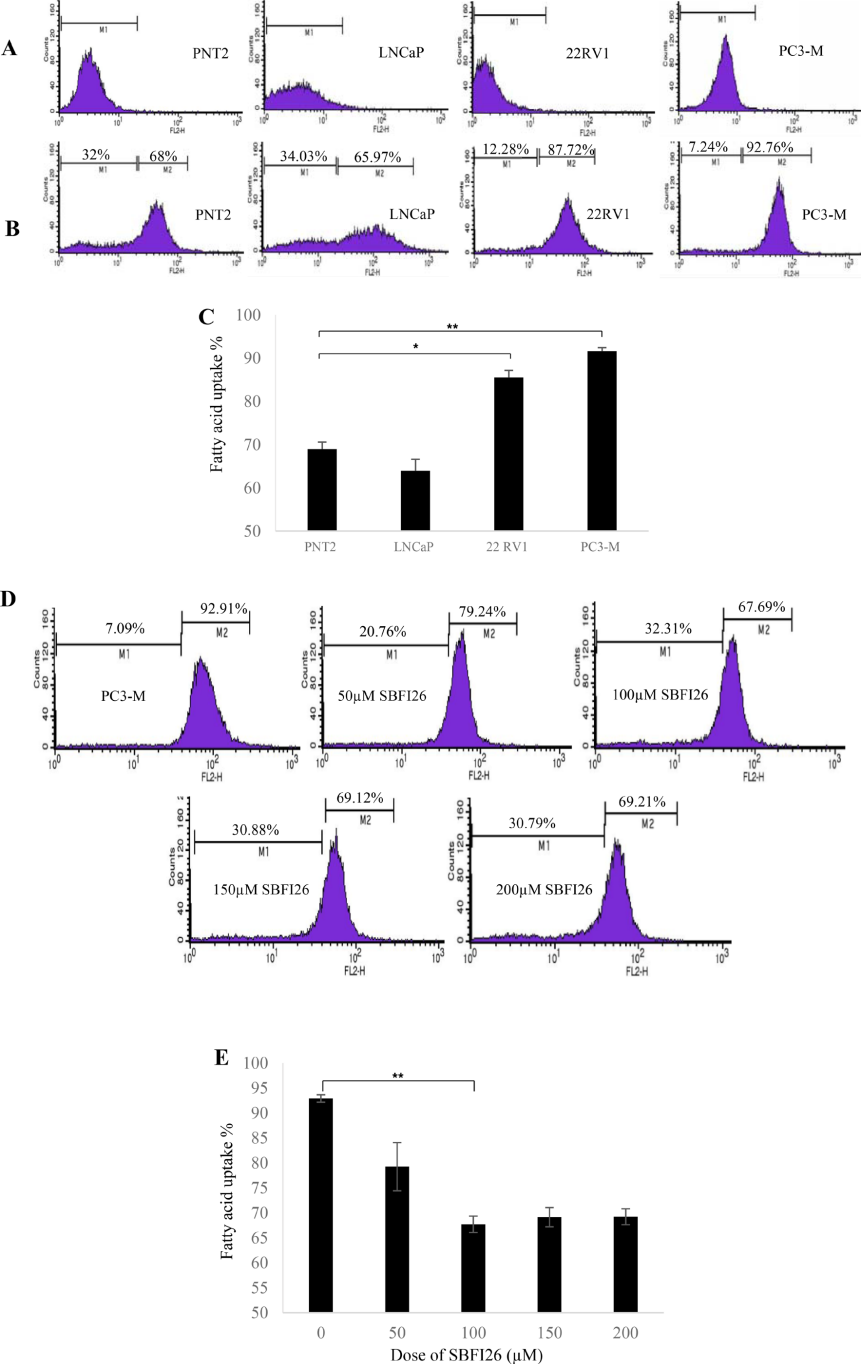
Fatty acid uptake of different prostate epithelial cell lines and inhibitory effect of SBFI26 in PC3-M cells (**A**) Representative histograms for unstained PNT2, LNCaP, 22RVI and PC3-M cells without adding BODIPY-labelled fatty acid. The marker M1 highlights negative peaks of the subclass control. (**B**) Representative histograms for fluorescence of stained PNT2, LNCaP, 22RVI and PC3-M cells 30min after adding BODIPY-labelled fatty acid and the marker M2 is placed to the right of M1 to highlight positive events (total percentage of cells with BODIPY-labeled fatty acid). (**C**) Percentages of cells taking up BODIPY-labelled fatty acid from different prostate epithelial cell lines. (**D**) Representative histograms for fatty acid uptake of PC3-M cells at a fixed concentration of BODIPY-labelled fatty acid with different concentrations of SBFI26. M1, unstained cells; M2, stained cells. (**E**) Percentages of cells with fatty acid uptake from PC3-M control (untreated) and those treated with different concentrations of SBFI26 for 30min with a fixed concentration of BODIPY-labelled fatty acid. Fluorescence intensity of each cell line was measured with an EPICS XL Cytometer (Beckman) at 570 nm and data analysis was performed with SYSTEM II™ Software. Values were plotted as mean ± SE (error bars). The differences between the control and the experimental groups were assessed by 2-tailed unpaired Student’s *t* test. *P < 0.01; **P < 0.001.

To investigate possible effect of FABP5 inhibitors on fatty acid uptake of PC3-M cells, a fatty acid uptake assay was performed using red fluorescence-labelled fatty acid BODIPY (Figure 5). Unstained cells (without BODIPY) were present in M1 zone (Figure 5A) and BODIPY stained cells were present in M2 zone after 30min incubation (Figure 5B). In contrast to benign PNT2, significantly more than 20% and 25% of cells took up fatty acid in moderately malignant 22RV1 and highly malignant PC3-M (Student’s *t* test *p* < 0.01 and *p* < 0.001) cells, respectively. Levels of fatty acid uptake between benign PNT2 and weakly malignant LNCaP cells were similar (Figure 5C). The effect of increasing concentration of SBFI26 on fatty acid uptake in PC3-M cells was determined using a fixed concentration of BODIPY (Figure 5D). When inoculated with SBFI26, cellular fatty acid uptake into PC3-M cells was reduced from 92.9% in the control in a dose-dependent manner, the maximum reduction with 100 μM was 67.7% (Figure 5E).

### SBFI26 inhibited PPARγ activation

**Figure 6:**
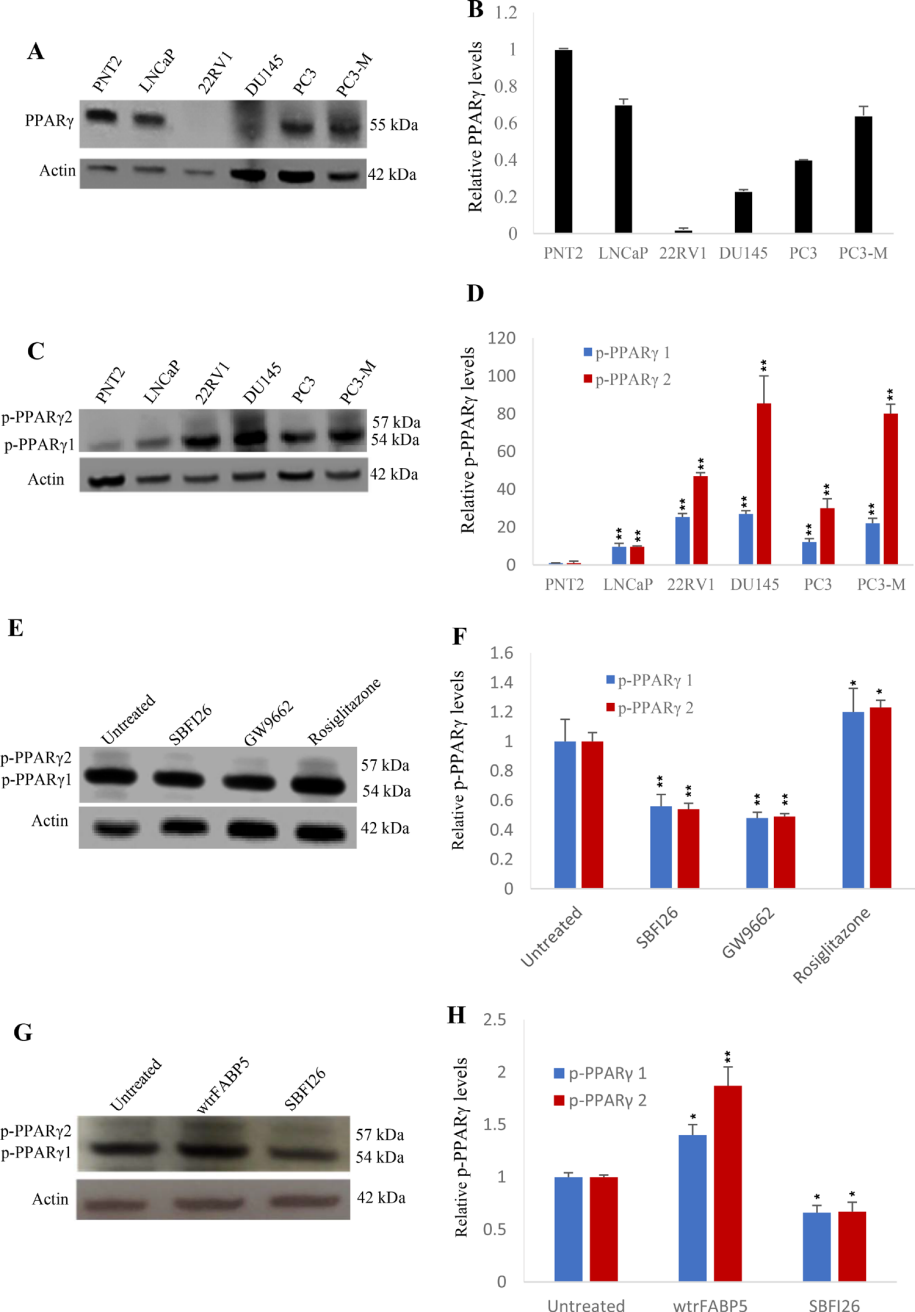
Effects of SBFI26 on levels of biologically active PPARγ or phosphorylated PPARγ (p-PPARγ1 and p-PPARγ2) in prostate cancer cells (**A**) Western blot of PPARγ expression in benign and malignant prostate epithelial cells. (**B**) Quantitative assessment of levels of PPARγ in benign and malignant prostate epithelial cells. The level of PPARγ in the benign prostate PNT2 cells was set at 1; levels in the other prostate cell lines were obtained by comparison with that in PNT2. (**C**) Western blot analysis of p-PPARγ1 and p-PPARγ2 in benign and malignant prostate epithelial cells. (**D**) Quantitative assessment of the levels of p-PPARγ1 and p-PPARγ2 in prostate cells. Levels of p-PPARγ1 and 2 in benign PNT2 cells were set at 1; levels in the other prostate cells were obtained by comparison with those in PNT2. (**E**) Effect of 24 h treatments with SBFI26, PPARγ antagonist (GW9662) and PPARγ agonist (Rosiglitazone on levels of p-PPARγ1 and 2 in PC3-M cells. (**F**) Quantitative assessment of p-PPARγ1 and 2 levels in PC3-M cells after treatments with SBFI26, GW9662 and Rosiglitazone. Levels of both p-PPARγ1 and 2 in untreated PC3-M cells were set at 1; levels in the other treated cells were obtained by comparison with those in untreated PC3-M. (**G**) Effect of 24 h treatments with wtrFABP5 and SBFI26 on levels of p-PPARγ1 and 2 in 22RV1 cells. (**H**) Quantitative assessment of levels of p-PPARγ1 and 2 in 22RV1 cells. Levels of both p-PPARγ1 and 2 in the control were set at 1; levels in the other treated cells were obtained by comparison with those in controls. For each Western blot, anti-β-actin was incubated with the same blot to normalize for possible loading errors. Results (mean ± SE) were obtained from 3 separate experiments and the differences between the control and the treatments in each experiment were assessed by 2-tailed unpaired Student’s *t* test. *P < 0.05; **P < 0.001.

The effect of FABP5 inhibitor SBFI26 on levels of biologically active PPARγ or phosphorylated PPARγ (p-PPARγ1 and p-PPARγ2) in benign and malignant prostate epithelial cells is shown in Figure 6. Western blot detected a PPARγ band at 55kDa in most of the cell lines used (Figure 6A). When the level of PPARγ in PNT2 was set at 1.0, relative levels of PPARγ in LNCaP, 22RV1, DU145, PC3 and PC3-M were 0.70 ± 0.03, 0.02 ± 0.01, 0.22 ± 0.001, 0.4 ± 0.0 and 0.64 ± 0.04, respectively (Figure 6B). When Western blot was used to detect p-PPARγ, 2 bands representing isoforms of p-PPARγ1 and p-PPARγ2 were found at 54 and 57kDa, respectively (Figure 6C). If levels of p-PPARγ1 and p-PPARγ2 in PNT2 were set at 1 and 1, relative levels in LNCaP, 22RV1, DU145, PC3 and PC3-M were 9.54 ± 1.81 and 9.5 ± 0.5; 25.4 ± 1.8 and 47.0 ± 1.7; 26.99 ± 1.72 and 85.5 ± 14.5; 12.08 ± 1.8 and 30 ± 5; and 21.99 ± 2.63 and 80 ± 5, respectively (Figure 6D). Levels of p-PPARγ, particularly p-PPARγ2, were significantly increased in all malignant cell lines (Student’s *t* test, *p* < 0.001). To investigate the effect of FABP5 inhibitor SBFI26 on p-PPARγ, PC3-M cells were treated with SBFI26, GW9662 and PPARγ agonist Rosiglitazone for 24 hours (Figure 6E). If levels of p-PPARγ1 and p-PPARγ2 in untreated cells were set at 1 and 1, the levels after treatment with SBFI26 and GW9662 were reduced significantly by 44% and 46%; 52% and 51%; respectively (Student’s *t* test, *p* < 0.001). However, in those cells treated with rosiglitazone, significantly increased levels of both p-PPARγ isoforms were observed (Student’s *t* test, *p* < 0.01) (Figure 6F). When treatments with wtrFABP5, SBFI26 were tested in androgen-sensitive 22RV1 cells (Figure 6G), wtrFABP5 significantly increased levels of both p-PPARγ1 and 2 (Student’s *t* test, *P* < 0.01) (Figure 6H). But treatment with the inhibitor SBFI26 suppressed the levels of both p-PPARγ isoforms.

## DISCUSSION

Since it was discovered that prostate cancer cell growth is dependent on the promoting effect of male hormone supplied through peripheral blood circulation [23], ADT targeting AR and circulating androgen has been the main therapeutic method to treat prostate cancer patients during the past 4 decades. However, the disease relapses within a period of time with a more aggressive form, called androgen-independent prostate cancer or castration resistant prostate cancer which does not respond to ADT effectively anymore. The conversion of androgen-dependent cancer cells to androgen independent CRPC cells is a fundamental change and the molecular mechanisms involved in this change is not fully known. Currently, there are a number of different hypotheses on how the androgen-dependent cells were transformed to androgen-independent cells. The main theory is that the biological sensitivity of AR is amplified after the first round of ADT to such an extent that even micro-quantities of remaining hormone in peripheral blood can still promote the malignant progression of CRPC cells [24]. Thus further ADT on CRPC was a general clinical practice. However, some opposite opinions to this practice were proposed recently [25]. Our previous work suggested that AR may not be relevant to malignant progression of CRPC and that targeting FABP5- PPARγ-VEGF axis, rather than the AR-mediated signalling pathway, which was gradually replaced by the FABP5-related pathway as the reduced androgen-dependency, could be a more effective way for CRPC treatment [7]. Here for the first time, we targeted the oncogenic FABP5 and its related signal transduction pathway and successfully used a chemically-synthesized FABP5 inhibitor to treat CRPC in nude mice by suppressing the biological activity of FABP5.

A group of 4 chemical compounds capable of suppressing the transportation of anandamide by FABP5, was originally used as anti-nociceptive and anti-inflammatory agents. They worked by increasing levels of brain anandamide transported by FABP5 and produced analgesia [18, 19]. The DAUDA displacement assay showed that 3 of these 4 compounds had the ability to bind to wtrFABP5, just as well as the 3 fatty acids tested (Figure 1). The dissociation constant (*K*_d_) for titration of DAUDA with wtrFABP5 was within the range of the other FABPs [26]. Although Linoleic acid had the highest binding ability amongst the 3 fatty acids, SBFI26 inhibited the highest FABP5 binding ability amongst the 4 compounds, and thus was identified as the lead inhibitor of FABP5 for suppressing its fatty acid-binding ability. Androgen-independent prostate cancer cell line PC3-M, expressing high levels of FABP5 and PPARγ [7, 27] was an extremely malignant and metastatic cell line. When the biological function of FABP5 was inhibited using SBFI26, significant anti-proliferation, anti-invasive, anti-migration and anti-anchorage-independent growth *in vitro* using PC3-M cell line was observed (Figure 2). SBFI26 as a lead inhibitor of FABP5 showed efficient anti-tumour roles in the mouse model for primary tumours implanted in the prostate gland (by 4.9- fold) and inoculated in the flank (by 52%). Compared to the control group, in which all mice (100%) developed metastases, SBFI26 treatment suppressed metastases in half of the mice of the treated group (50%). These results suggest that SBFI26 can be used as an anti-tumour agent to treat CRPC.

Increased levels of FABP5 play a crucial role in promoting malignant progression in CRPC cells by binding and transporting increased amounts of fatty acids to stimulate PPARγ [7, 13, 28, 29]. It is known that increases in uptake of fatty acids will contribute to the switch in energy production from aerobic to anaerobic sources as well as the downstream effect of increased production of VEGF to stimulate angiogenesis. These changes are induced by increased levels of FABP5 and may contribute to the amelioration of the effects of chronic hypoxia which is known to occur as prostate cancer develops [30, 31]. In this work we showed that the treatment of PC3-M cells with the PPARγ antagonist, GW9662, produced a better suppression of tumour growth to that obtained by SBFI26 (Figure 4). This result suggests that the suppressive mechanisms of the inhibitor may be related to the FABP5- PPARγ- signal transduction pathway [7]. In confirmation, we showed not only the fatty acid uptake was increased with increasing malignancy of prostate cancer cells, but also that SBFI26 produced a remarkable reduction in fatty acid uptake into PC3-M cells (Figure 5). These results suggest that SBFI26 may be a competitive inhibitor for FABP5 and hence prevent intra- and extra-cellular fatty acids from being transported into the cytoplasm. The reduced fatty acid uptake produced by SBFI26 may result in a remarkable reduction or cessation of the stimulation of PPARγ by fatty acids. Thus PPARγ may no longer be able to upregulate the down-stream cancer-promoting genes, such as VEGF, and to suppress apoptosis [32, 33].

Our recent study showed that the FABP5-PPARγ-VEGF signalling transduction axis, not the androgen receptor-initiated pathway, is a dominant route for transduction of malignant signals in CRPC cells [7]. In this axis, the role of PPARγ is essential. Thus, although the total PPARγ expressed in the malignant cell lines was not higher than that in the benign PNT2 cells (Figure 6A, 6B), both biologically-activated PPARγ isoforms (or phosphorylated PPARγ) p-PPARγ1 and p-PPARγ2 [34, 35] increased with increasing cellular malignancy (Figure 6C, 6D). The p-PPARγ isoforms in PC3-M cells, which were expressed in high levels and were further increased by rosiglitazone (PPARγ agonist), their levels had greatly reduced by treatments with SBFI26 and GW9662 (Figure 6E, 6F). These results suggest that SBFI26 may act as an inhibitor to block the stimulation of fatty acids transported by wild type FABP5 and hence prevent activation of PPARγ. PPARγ is a fatty acid receptor localised in the nuclear membrane [36–38]. Thus the inhibition of phosphorylation by SBFI26 is likely to cause inhibition of fatty acid uptake. It has been suggested that SBFI26 is a weak agonist of PPARγ [18]. Since SBFI26 suppressed fatty acid uptake by replacing fatty acids which bind to FABP5, it is possible that some SBFI26 may be delivered to activate PPARγ in a much weaker way than with the fatty acids. This may be the reason why SBFI26 produced a slightly lower degree of suppression in tumourigenicity and metastasis than GW9662. Although SBFI26 did not produce a complete inhibition of CRPC, its therapeutic effect was highly significant.

PPARγ is highly expressed in adipose tissue and plays an important role to regulate adiposity and insulin sensitivity [39]. Two biologically active isoforms of PPARγ, PPARγ1 and PPARγ2, are expressed in human tissues [34, 35]. The potential of using PPARγ as a direct target for cancer treatment has been widely investigated during the past decade but still remains debatable. Both PPARγ agonist and antagonist have shown some anticancer effect through PPARγ-dependent and -independent pathway [40, 41]. However, there are safety concerns: side effects, including dose limited side effects linked to PPARγ drug treatments, increased the risk of cardiac failure and potential carcinogenicity in rodents [42]. In addition, PPARγ agonist inhibitors have been shown to suppress cell growth and induce apoptosis of prostate cancer cells by both PPARγ-dependent (genomic) and - independent (non-genomic) signalling pathways. Thus it remains unclear whether the non-genomic effects are essentially on PPARγ pathways [43]. GW9662 is a potent, irreversible and selective PPARγ antagonist, and it has been reported to inhibit growth of human breast cell line in a PPARγ- independent manner [44]. However, the results from another study indicated that GW9662 has a protective role in cancer by blocking cannabinoids-induced apoptosis in xenograft-induced tumours in mice [45]. It was found that GW9662 has a significant effect on adipose tissue weight and glucose metabolism *in vivo*. If GW9662 is administrated continuously for a long time, it can reduce weight and suppress any increase in the amount of visceral adipose tissue [46]. In addition, GW9662 upregulates the expression of several genes associated with the transcription, processing, splicing and translation of RNA [47]. Although our results in this study showed that GW9662 produced significant reduction in the sizes of tumours developed from cancer cells inoculated subcutaneously in flanks of the mice, using GW9662 as a therapeutic reagent is hardly possible because of its none-specificity. In fact, due to the versatile nature of PPARγ in its biological function, targeting PPARγ directly for cancer treatment is also difficult to achieve.

Previous work suggested that the dependency of the prostate cancer cells on the FABP5-related pathway was gradually increased with a correspondingly reduced dependency on the AR-initiated pathway until the former became completely dominant [7]. In this study, in androgen-responsive, moderately-malignant 22RV1 cells, SBFI26 produced a reduction in both PPARγ activated isoforms by an average of 33.5% (Figure 6G, 6H). This level of reduction was much lower than that caused by SBFI26 in the androgen-independent, highly malignant PC3-M cells (average reduction was about 50%). These results suggest that the proportion of activated PPARγ regulated by the FABP5-related pathway was much higher in PC3-M cells than that in 22RV1 cells, a result which suggests that treatment by suppression of the FABP5-pathway is more effective in CRPC cells. This supports the previous finding that ADT would lose its effect gradually as the cancer cell become more independent of androgen for growth and is consistent with the eventual loss of AR receptor in most advanced cancers [7, 25].

In summary, we have targeted the FABP5-PPARγ signalling pathway by suppressing the biological activity of oncogenic FABP5 so that the signalling molecules fatty acids cannot be passed to PPARγ. Thus this signalling axis is ceased to functioning due to the lack of fatty acids stimulation. Therefore, the FABP5 inhibitor SBFI26 supressed the malignant progression of CRPC by cutting off the FABP5-related signalling transduction chain at the initial stage and it may be a candidate reagent for a CRPC treatment.

## MATERIALS AND METHODS

### Cell lines and chemical inhibitors

The benign cell line PNT2 [48], highly malignant, androgen-independent cell lines DU145 [49], PC3 [50] and PC3-M [51], the moderately malignant, androgen-responsive cell line 22RV1 [52], and the weakly malignant cell line LNCaP [53] were cultured and maintained in 1640 medium (Invitrogen) supplemented with 10% FCS (Biosera), 100 U/mL penicillin and 100 μg/mL streptomycin (Invitrogen). For LNCaP cells, 100 μg/mL sodium pyruvate (Sigma) was added to the culture medium. Chemically synthesized FABP5 inhibitors used in this study, including SBFI26 (cat# 8009-2334), SBFI19 (cat# 5511-0235), SBFI27 (cat# 8009-7646) and SBFI31 (cat# C075-0064) were purchased from ChemDiv, dissolved in DMSO, and stored at –20°C. The working concentration of DMSO for all *in vitro* assays was 0.1% (v/v).

### Ligand binding assay

The fatty acid-binding ability of wtrFABP5 was examined by using the DAUDA displacement assay which used fatty acids and different chemical compounds to replace the fluorescently labelled fatty acid analogue DAUDA (Cayman). The dissociation constant (*K*_d_) of wtrFABP5 was measured by titrating different concentrations of DAUDA (0.4–3 μM) to a solution of 3 μM wtrFABP5 in PBS. For calculation of *K*_d_ values, the excitation and emission wavelengths used were 345 and 530 nm, respectively. For each experiment, the fluorescence data were normalized to the peak fluorescent intensity [54], and then subtracted from the data of samples without protein. The data were fitted by nonlinear regression using GraphPad Prism software to a saturation binding curve model to estimate the apparent dissociation constant (*K*_d_) and maximal fluorescence intensity (*B*_max_). The inhibition constant (*K*_i_) was measured to determine the potency of different fatty acids (Linoleic, Oleic, Palmitic acid) (Sigma) and different inhibitors (SBFI26, SBFI19, SBFI27, and SBFI31 to wtrFABP5 by their ability to displace DAUDA. Three μM wtrFABP5 was incubated with 2 μM DAUDA in PBS in the presence or absence of each fatty acid or each chemical inhibitor in different concentrations (0.5–20 μM). Loss of fluorescence intensity was measured with Varioskan Flash and the data were fitted by nonlinear regression using GraphPad Prism software to a one site binding affinity model to estimate the binding affinity. The *K*_i_ of each ligand was determined using the equation *K*_i_ = *IC*_50_/1+ (DAUDA concentration /*K*_d_). The lead compound and the best fatty acid that produced the highest binding affinity were then added to the assay at 10 μM, and tested in triplicate to confirm their activity.

### Cell viability and proliferation assay

PC3-M cells (5 × 10^4^) were plated in triplicate in 96 well plates and incubated overnight. Cells were treated with different concentrations of SBFI26 (25–125 μM) for 24 h. Cell viability was assessed using MTT assay, as described previously [12]. The anti-proliferative effect of the best concentration for SBFI26 was determined after 6 days of treatment.

### Migration assay

Wound healing migration assay was carried out to evaluate the effect of SBFI26 on the migratory rate of PC3-M cells. Wounds were generated by scratching the monolayer cells with a blue pipette tip. The floating cells were removed by washing with PBS and inhibitors were added to the culture medium. The wound was photographed under the microscope at 0, 12 and 24 h after treatment and the wound widths were assessed by quantitative analysis using ImageJ software.

### Invasion assay

PC3-M cells in serum-free medium were seeded in the upper Boyden chamber (BD Biosciences) in triplicate at a density of 2.5 × 10^4^ cells per well in serum-free medium. Complete medium was added to the lower chambers. After 3 hours of incubation, 100 μM SBFI26 was added to the upper chambers. After 24 h of incubation, cells that invaded the lower chambers were stained with crystal violet and counted with a cell counter.

### Soft-agar assay

Low melting agarose was seeded in 6-well plates and 5 × 10^4^ cells/well layered on agar, followed by 200 μl of medium alone or medium with FABP5 inhibitor SBFI26. Colonies larger than 300 μm in each well were counted 2 weeks later in a similar way to that described previously [12].

### Nude mouse assay to test tumorigenicity and metastasis

PC3-M cells were transfected with the pGL4.50 [*luc2*/CMV/Hygro] vector (Promega) using FuGene HD transfection reagent (Promega) following the manufacturer’s instructions. Individual colonies were isolated by ring cloning and 3 colonies that stably-expressed the highest bioluminescence signals were identified using D-luciferin (Promega) with a Varioskan Flash Reader (Thermo Scientific). Association of the luminescence intensity with the number of cells was assessed by an IVIS imaging system (Perkin Elmer). Cells (5 × 10^5^) from PC3-M- *luc2* colony were suspended in 30 μL PBS and orthotopically implanted into the dorsal prostate of 33 male Balb/c nude mice (Charles River, UK) (8–10 weeks old), as described previously [55]. One week later, tumour-bearing mice were divided into 2 groups (8 each) and subjected to the following intraperitoneally injections: 1) control with PBS; 2) SBFI26, 1mg/kg. Injections were repeated every two days for 25 days and the metastatic loci were monitored weekly using the IVIS after mice were subcutaneously injected with D-luciferin (150 mg/kg). Bioluminescence images was analysed using the Living Imagine software (Xenogen) and the measurement recorded was based on total photons/second (p/s) within each defined region of interest.

### Nude mouse tumorigenecity assay

PC3-M cells (2 × 10^6^) in 200 μL PBS were subcutaneously injected into the right flank region of the mouse (6–8 week old) to test the suppressive effect of the inhibitors on tumorigenicity. In the first round, 3 groups of mice (8 each) were used: 1) control with PBS; 2) 1mg/kg SBFI26, injected from the 1^st^ day after cell inoculation; 3) 1mg/kg SBFI26, injected from the 7^th^ day after cell inoculation. In the second round, 2 groups of mice (5 each) were used and at 7 days after the cell inoculation, each group was subjected to different intra-tumoural injections: 1) control with PBS; 2) PPARγ antagonist (GW9662, 1mg/kg) (Sigma). The injections were repeated every 2 days for 30 days, tumour size was measured every 3–4 days and the volume calculated by the formula of L× W×H×0.5236 [56]. Work was performed in accordance with UKCCCR guidelines under Home Office License PPL40/2963.

### Fatty acid uptake assay

Assay for fatty acid uptake was performed using red fluorescence-labelled BODIPY [57]. The fluorescence intensity from cells before and 30 minutes after adding BODIPY was measured to determine fatty acid uptake. In inhibition and competition experiments, different concentrations of unlabelled lead compound SBFI26 (50–200 μM) with the same concentration of labelled BODIPY were added directly to the highly malignant PC3-M cells.

### Statistical analysis

Student’s *t*-test was carried out using GraphPad Prism software to compare the differences of the means between control and experimental groups. All *in vitro* experiments were conducted in triplicate and repeated at least three times. The difference is regarded as significant when *p* < 0.05; in the results, *p* value is represented by asterisks as follows: *P < 0.05; **P < 0.001; ***P < 0.0001.
