# Correction: Inhibitor SBFI26 suppresses the malignant progression of castration-resistant PC3-M cells by competitively binding to oncogenic FABP5

**DOI:** 10.18632/oncotarget.27690

**Published:** 2020-08-04

**Authors:** Waseem Al-Jameel, Xiaojun Gou, Shiva S. Forootan, Majed Saad Al Fayi, Philip S. Rudland, Farzad S. Forootan, Jiacheng Zhang, Philip A. Cornford, Syed A. Hussain, Youqiang Ke

**Affiliations:** ^1^ Molecular Pathology Laboratory, Department of Molecular and Clinical Cancer Medicine, Liverpool University, Liverpool, L3 9TA, United Kingdom; ^2^ Sichuan Antibiotics Industrial Institute, Chengdu University, Chengdu 610081, China; ^3^ Department of Biochemistry, Liverpool University, Liverpool, L69 3GA, United Kingdom

Original article: Oncotarget. 2017; 8:31041–31056. 31041-31056. https://doi.org/10.18632/oncotarget.16055

**This article has been corrected:** The following references were accidentally omitted from the published list:
